# Requirements analysis for an AI-based clinical decision support system for general practitioners: a user-centered design process

**DOI:** 10.1186/s12911-023-02245-w

**Published:** 2023-07-31

**Authors:** Dania Schütze, Svea Holtz, Michaela C. Neff, Susanne M. Köhler, Jannik Schaaf, Lena S. Frischen, Brita Sedlmayr, Beate S. Müller

**Affiliations:** 1 Goethe University Frankfurt, Institute of General Practice, Theodor-Stern-Kai 7, 60590 Frankfurt, Germany; 2 Goethe University Frankfurt, University Hospital, Institute of Medical Informatics, Frankfurt, Germany; 3 Executive Department for Medical IT-Systems and Digitalization, University Hospital Frankfurt, Goethe University, Frankfurt, Germany; 4 Technische Universität Dresden, Institute for Medical Informatics and Biometry, Carl Gustav Carus Faculty of Medicine, Dresden, Germany; 5 University of Cologne, Faculty of Medicine and University Hospital Cologne, Institute of General Practice, Cologne, Germany

**Keywords:** Clinical decision support systems, Computer-assisted diagnosis, Primary care, User-centered design, Qualitative research, Requirements analysis

## Abstract

**Background:**

As the first point of contact for patients with health issues, general practitioners (GPs) are frequently confronted with patients presenting with non-specific symptoms of unclear origin. This can result in delayed, prolonged or false diagnoses. To accelerate and improve the diagnosis of diseases, clinical decision support systems would appear to be an appropriate tool. The objective of the project ‘Smart physician portal for patients with unclear disease’ (SATURN) is to employ a user-centered design process based on the requirements analysis presented in this paper to develop an artificial Intelligence (AI)-based diagnosis support system that specifically addresses the needs of German GPs.

**Methods:**

Requirements analysis for a GP-specific diagnosis support system was conducted in an iterative process with five GPs. First, interviews were conducted to analyze current workflows and the use of digital applications in cases of diagnostic uncertainty (as-is situation). Second, we focused on collecting and prioritizing tasks to be performed by an ideal smart physician portal (to-be situation) in a workshop. We then developed a task model with corresponding user requirements.

**Results:**

Numerous GP-specific user requirements were identified concerning the tasks and subtasks: performing data entry (open system, enter patient data), reviewing results (receiving and evaluating results), discussing results (with patients and colleagues), scheduling further diagnostic procedures, referring to specialists (select, contact, make appointments), and case closure. Suggested features particularly concerned the process of screening and assessing results: e.g., the system should focus more on atypical patterns of common diseases than on rare diseases only, display probabilities of differential diagnoses, ensure sources and results are transparent, and mark diagnoses that have already been ruled out. Moreover, establishing a means of using the platform to communicate with colleagues and transferring patient data directly from electronic patient records to the system was strongly recommended.

**Conclusions:**

Essential user requirements to be considered in the development and design of a diagnosis system for primary care could be derived from the analysis. They form the basis for mockup-development and system engineering.

**Supplementary Information:**

The online version contains supplementary material available at 10.1186/s12911-023-02245-w.

## Background

General practitioners (GPs) are the first point of contact for patients with health issues in many countries. Frequently, patients consult their GPs with ambiguous symptoms of unknown origin [1]. Although having a common disease, such patients may also present with atypical or non-specific symptoms [2]. Furthermore, symptoms may vary between men and women, which can lead to diagnostic uncertainty [3–6]. At the same time, patients with ambiguous symptoms may be suffering from a rare disease [2, 7]. According to current estimates, 5,000 to 8,000 rare diseases exist [8, 9]. The European Union considers a disease as rare when it affects no more than 1 in 2,000 persons and estimates that about 30 million people in Europe are affected [9]. Diagnosing a rare disease can often take years, during which uncertainty, emotional and physical burden, false diagnoses and numerous physician consultations are the norm [7, 10–12] and even after years, many cases remain unclear [13]. When symptom complexes are ambiguous or unfamiliar to the physician, clinical decision support systems (CDSS) can help GPs form a diagnosis and make a decision [14–16]. According to Sim et al., a CDSS is defined as ‘software designed to be a direct aid to clinical decision-making, in which the characteristics of an individual patient are matched to a computerized clinical knowledge base and patient-specific assessments or recommendations are then presented to the clinician or the patient for a decision’ [17]. Several CDSS are used in primary care, mainly for screening and diagnosing common chronic diseases. Research shows that CDSS have the potential to improve and accelerate the diagnosis [14]. However, little is known about the use of CDSS for acute and uncommon diseases in primary care [14]. Studies have identified several barriers and challenges to CDSS and their use, such as poor workflow integration, a lack of acceptance or trust, and poor usability [14, 15, 18–21]. To address these barriers and establish CDSS as a means of helping GPs form a diagnosis when confronted with unclear disease patterns in primary care, a user-centered approach needs to be taken in the development of future systems [14, 20–22].

The objective of the ‘Smart physician portal for patients with unclear disease’ (SATURN) project funded by the German Federal Ministry of Health, is to develop an Artificial Intelligence (AI)-based diagnosis support tool for GPs. The medical focus thereby is on making a diagnosis in cases of diagnostic uncertainty. On a technical level, rule-based systems, machine learning, and case-based reasoning will be employed. A rule-based system can be used to make a diagnosis based on guidelines. Machine learning allows a diagnosis to be predicted with a statistical probability, while case-based reasoning identifies similar patient cases from a case base and presents them to the user. Existing CDSS generally use only one method of decision support [22].

To ensure that the diagnosis tool meets the needs of future users, is widely accepted, and shows a good usability, GPs will be continuously involved in the user-centered design (UCD) process. UCD is an engineering strategy that, when developing interactive systems, investigates and focuses on the needs, desires, and limitations of end users. Information on the context of use is therefore collected, and an analysis of user requirements is carried out [23–28].

In this paper, we present the user requirements analysis we conducted with GPs as the first step in the development of an AI-based CDSS in German primary care. The objectives of the requirements analysis were (1) to investigate diagnostic workflows and (2) to find out what a new CDSS must provide from the user’s perspective.

## Methods

### Design

As part of the UCD, we used a qualitative design to conduct requirements analysis with a group of general practitioners [27, 29]. We carried out interviews to gain insight into current workflows (as-is situation) [26, 29, 30], and conducted a workshop to collect ideas, and identify needs and user requirements for a new CDSS for GPs (to-be situation) [31]. As a result, we developed a task model and identified user requirements for the new system. A task model is a summary of the tasks and subtasks that users will perform with the support of the new or revised interactive system [29]. User requirements describe what an interactive system should enable users to do in order to achieve their goals [29, 32].

**Fig. 1:**
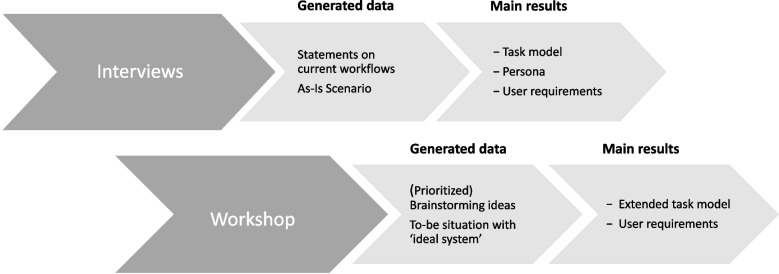
Design of requirements analysis and results

In the following sections, we describe the process in detail. Figure 1 shows the different steps of collecting data and obtained results.

The study was performed and reported in accordance with the Consolidated Criteria for Reporting Qualitative Research (COREQ) [33].

### Setting and sampling

First, the project team jointly defined and roughly characterized the target group for the CDSS. We then used a purposeful sampling approach [34] to constitute a group of five general practitioners to accompany the entire project. This group size is common because it already enables a high degree of usability to be achieved. This is especially true when the project is expected to involve multiple iterations [35–37]. We recruited male and female physicians and ensured they had a basic understanding of digitization. The participants were known to us through previous research projects on other topics. They were contacted by email, received written information on the study and provided their written informed consent.

### Data collection

#### Interviews

A team of researchers from the fields of sociology, medicine, medical sciences and medical informatics prepared an interview guide addressing the research question ‘How do general practitioners currently deal with cases of patients with ambiguous symptoms of unknown origin?’ The guide was presented in an interdisciplinary research group for qualitative methods at the Frankfurt Institute of General Practice, where it was discussed and adapted. The interview guide was also tested in three pretest interviews. The final guide (see Additional file 1) contained questions on the following topics: 1. Current workflow in cases of diagnostic uncertainty, 2. Use of digital applications in cases of diagnostic uncertainty, 3. Experience in the diagnosis of rare diseases, 4. Additional comments. Moreover, information was collected on age, number of years working in general practice, additional qualifications, employment status and the technical equipment available in the practice.

From March to May 2022, a researcher (SH) with a medical background and experience in qualitative methods interviewed the five participants by telephone. The length of the interviews ranged from 24 to 44 min. All interviews were audio-taped and transcribed in a paraphrased form. Selected passages were transcribed verbatim as quotations. Participants provided their verbal consent to be audio recorded at the beginning of the interview.

#### Workshop

In June 2022, we conducted a workshop with all five participating GPs to gather ideas and find out about user needs for a new CDSS for general practitioners. The workshop was performed online via Zoom and lasted for two hours. It was hosted by three study team researchers from the fields of medicine (SH), medical informatics (MN), sociology and medical sciences (DS). One researcher moderated the discussion, one took notes on the shared Zoom-whiteboard and one provided background information on the project, took notes and provided technical support. The participants were first told about the current status of the project, as well as the aim and structure of the workshop. We then conducted a brainstorming on the question: ‘What tasks would you like to perform with an ideal smart physician system for unclear diagnoses?’ The answers (ideas) were numbered and documented on the shared Zoom-whiteboard.

After the brainstorming, the participants were asked to name their top 5 ideas in the form of a template [37] that we provided via email. The template consisted of the following fields: 1. The number of the idea, 2. The name of the idea, 3. A brief description of the idea, and 4. Why this idea was important to them. All participants returned the completed template with their prioritized ideas by email. We ended the workshop with an opportunity for questions and a preview of the next steps in the project.

### Data analysis

#### Interviews

Three researchers (SH, MN, DS) transcribed the interviews in paraphrased form and analyzed them [29]. Each interview was first paraphrased by one person. A second researcher listened to the interview, reviewed the transcript and made additions where necessary. Following the method described by Geis and Polkehn [27, 29], we first derived user needs from the transcripts. These were phrased according to the following syntax rule: *The user must have* < *resource/information/…* > *to* < *make decision* > *or* < *execute action* > . In the next step, we formulated user requirements based on the user needs, describing what users must be able to do with the system: *The* < *user* > *needs to be able to* < *recognize/enter/select/…* > *in the system*. Table 1 shows an example. Thereafter, all results were checked and discussed by the study team and an overall list of user requirements was compiled (see Additional file 2). Finally, the user requirements were structured by identifying tasks and subtasks they referred to. These formed an initial task model that outlined the as-is situation [29]. In addition, a so-called proto-persona for the primary target group of ‘general practitioners’ was developed from assumptions and existing knowledge about the target group, and supplemented with details from the interviews. The persona aimed to create a uniform understanding of the target group in the project team in order to better prioritize development goals later on [38, 39].

**Table 1:** Example for derivation of user requirements from interviews

Paraphrased part of the interview	User need (N)	User requirement (UR)
In a case of suspected Fabry-disease, GP02 performed tests that she researched and ordered on the Internet herself. Her suspicion was ultimately not confirmed	N: The physician needs to know what tests to perform to rule out or confirm a diagnosis	UR: The physician needs be able to recognize in the system what tests he/she needs to confirm a suspected diagnosis
When GP05 looks at the results of a Google search, much of what is suggested has already been ruled out by examinations. GP05 then looks at what remains, and what has not yet been investigated but that seems reasonable	N: The physician needs to be able to sort suspected diagnoses based on which diagnoses have already been ruled out	UR: The physician needs to be able to exclude diagnoses in the system

#### Workshop and synthesis

As in the case of the interviews, we derived user requirements from the user needs expressed in the workshop with respect to a to-be situation and added them to the list of requirements. As some needs led to new tasks, we extended the task model so that it now represented the to-be situation.

## Results

### Participants

Five GPs participated in the requirements analysis. The characteristics of the participants can be found in Table 2.

**Table 2:** Participant characteristics

ID	Age	m/f	Additional qualifications or special focus	Time working in general practice	Employment status
1	43	m	Emergency care, focus on geriatrics and palliative care	9 years	self-employed
2	44	f	Intensive and emergency care	6 years	employed
3	33	m	None	9 months	employed
4	49	m	Diabetologist, nephrologist, focus on hypertensiology and nutritional medicine	8 years	self-employed
5	35	f	Focus on rare diseases	2.5 years	employed

### User group

Even though the project was designed with GPs in mind, the inclusion of healthcare assistants as a second user group would have been conceivable. However, it became apparent during the interviews that healthcare assistants were not part of the diagnostic workflow and thus would not be users of the system. The persona (Additional file 3) therefore represents the main user group of general practitioners.

### User requirements

**Fig. 2:**
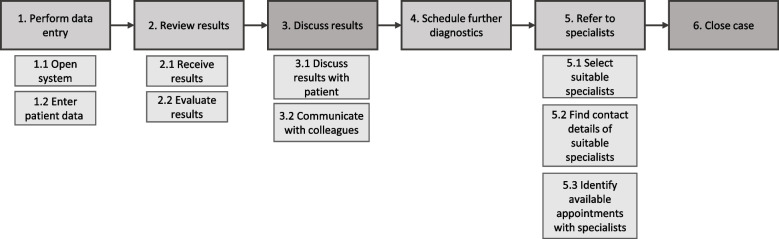
Task model for use of CDSS by GPs

In the interviews, the GPs described how they currently proceed in cases of diagnostic uncertainty and what digital and non-digital support options they typically use (including their advantages and disadvantages), as well as what they are currently missing. In the workshop, we collected ideas for a new CDSS. We created a task model according to the workflow and assigned requirements to it. The task model initially represented the results of the interviews and was later supplemented by the results of the workshop (see Fig. 2; steps 3 and 6 were added after the workshop). In the following, we report on the most important user requirements for each step of the task model. For better readability, we do not use the syntax employed in the analysis in the presentation of results, but report the requirements narratively and substantiate them with quotes from the interviews (I), the workshop (WS) and the Top 5 booklets provided by the participants.

#### Perform data entry

In the interviews, it became clear that basic requirements would have to be fulfilled before physicians could use the system at all. In general, it should be possible to use the system on different devices and with different operating systems. Physicians also need to be able to use it in parallel with practice management software and other colleagues.

The GPs named various essential data entry options for test results and information that they use for research, or that they would like to use in the future. These were: medical findings, vague descriptions of complaints, tentative diagnoses, family anamneses, laboratory parameters, symptoms and especially combinations of symptoms:

*Well what I think would probably be useful – […] you can directly enter a number of symptoms together – that’s rather practical of course because diagnoses generally differ as a result of specific symptom combinations. (I, GP3)*

Furthermore, the user needs to be able to enter the parameters weight, age and gender, as such information is essential when making a diagnosis:

*Well the combination of ‘[…] I have a symptom, which might even be a leading symptom and well, I can filter out the probable diagnoses according to sex, age, or age group‘. (I, GP3)*

In this regard, it became evident that GPs would much prefer to simply transfer sociodemographic and patients’ medical data from the practice management system to the CDSS rather than enter them manually, as it would save time and reduce possible transmission errors.

Another outcome of the workshop was that GPs would like to create patient records in the system, which they could then access and rework at any time:

- Excerpt from a Top 5 booklet:
- Name of the idea:

*Create patient record that is possible to update.*

- Description of the idea:

*Save information that has already been entered so that you can continue to work on it later.*

- Why is this important to you?

*So that I can work on the case again and again and add new information later; to save me work (to avoid doing the same thing over and over again). (Top 5 booklet, GP2)*

The participants also pointed out that in a best case scenario, suggestions for entries would be made automatically during the data entry process, perhaps in the form of an intelligent query, which would help in recognizing what further data and test results should be entered.

#### Review results

An important step that was highlighted in the interviews was the screening and assessment of results. GPs emphasized that in a primary care setting, the CDSS should not focus exclusively on rare diseases, but should also consider common diseases. This became obvious in the criticism of a ‘symptom checker’ that already existed:

*The end result is that the extent of a symptom match with certain diagnoses is given in percentage terms. And then my feeling was that the usual diagnoses you make in family practice didn’t play a role but that it was more like rare diseases were matched. I would say that our classic [diagnoses], the ones that occur a lot – there wasn’t really a match for them. (I, GP1)*

To assess the results, it was essential for the participants to see which differential diagnoses should be considered:

*When I'm stuck with a patient, I would just like a portal with a straightforward user interface that permits me to simply enter symptoms and medical findings or diagnoses as keywords so that the system ultimately just provides me with differential diagnoses. (WS, GP4)*

At the same time, the GPs would like to be able to manually hide diagnoses that have already been ruled out by previous tests from the list of results:

- Description of the idea:

*In the results section, I would like to be able to ‘gray out’ all suggested diagnoses that have already been ruled out.*

- Why is this important to you?

*Leads to more clarity—one could focus on the remaining diagnoses. The system could learn from this. (Top 5 booklet, GP5)*

In order to be able to evaluate the results, participants recommended that the probability of specific diagnoses be provided:

- Description of the idea:

*There are different ways in which search results could be sorted, e.g. alphabetically, by date of inclusion in the database or similar. The most sensible way would be a ‘best-match’ for the given search parameters, if possible with an indication of probability.*

- Why is this important to you?

*Makes it easier to focus on the most relevant diagnoses of all the different possibilities. (Top 5 booklet, GP3)*

In addition, the participants thought the user needed to be able to identify the source of the provided information:

- Description of the idea:

*Details on the source of [the information on] a particular diagnosis that the system provides should be provided, along with the guideline/expert knowledge, *etc*., on which the portal is based.*

- Why is this important to you?

*Transparency, and so I can check whether I consider the source to be reliable. It might also be important to re-read it. (Top 5 booklet, GP5)*

Ideally, users should also have free access to the provided information.

#### Discuss results

In the workshop, it became clear that the GPs would like to be able to share their initial thoughts and results with patients and colleagues. GPs wanted the system to provide a platform through which they could communicate with colleagues when cases were unclear:

- Description of the idea:

*It should be possible to communicate live—in a kind of forum—with colleagues, experts should also participate and contribute to the solution of the problem.*

- Why is this important to you?

*The AI is refined –‘fed’–with NI (Natural Intelligence). (Top 5 booklet, GP4)*

In this context, some participants raised the idea of giving patients access to their own patient records in the CDSS:

*Just so the patient has the chance to follow what’s going on, like when patients are transferred to specialists who then receive a copy of the report or something like that. […] So that they can also intervene and correct certain things - if he sees that I have described something differently to the way he would, perhaps the symptoms, for example. (WS, GP4)*

Other participants were critical of this suggestion and recommended restricting access:

*If patients did have the chance to enter data or symptoms themselves, I think it would be important that it was possible to activate or dis-activate patient access because when I think of certain patients, who get completely carried away and can’t focus at all, which can make consultations very confusing […] That‘s why I wouldn’t like it if every patient had access. (WS, GP2)*

#### Schedule further diagnostics

When the system has generated several results, users need to be able to confirm or rule out differential diagnoses. For example, the GP might need information on which tests he or she would need in order to diagnose a rare disease:

*A further step that I think would be rather nice would be to be told which diagnostic procedures could be used to confirm or rule out the differential diagnoses that the system identifies because I thought that was a big problem with ADA Health [a symptom checker app]. You‘re shown at the end, in percentage terms, what diagnoses could explain it, but for me, one step further would be to receive an indication, okay, how can I best confirm or rule out the diagnosis. (WS, GP4)*

#### Refer to specialist

When the GPs would like to refer the patient to a specialist for further diagnostic tests, they would like the system to recommend the most appropriate physician to contact for a specific suspected diagnosis. If suitable information platforms already exist for this purpose, the user should be forwarded to them:

- Description of the idea:

*The portal should link to existing solutions, e.g. SE-Atlas [Care atlas for people with rare diseases], Orphanet, *etc*.*

- Why is this important to you?

*You don't have to reinvent everything. Resources that already exist should be used and linked to. If the portal is widely used, it might increase the visibility of existing structures like SE-Atlas. (Top 5 booklet GP1)*

In addition, participants ideally wanted to be able to use the system to make direct, uncomplicated contact to experts in the relevant field.

#### Close case

On closing a case, GPs would like to be able to enter confirmed diagnoses into the system, as it would enable colleagues to benefit and learn from their experience. The participants recommended that completed cases should be made available in an edited form, so that other users could access them:

*When the case has been completed, then the possibility to describe it [would be useful], that is to say to see the process, how the whole thing developed and what actually came out of it all in the end. (WS, GP4)*

## Discussion

Our requirements analysis was the first step in an UCD process to develop a CDSS for primary care. With the help of interviews and a workshop we identified user needs and determined user requirements from the GP’s perspective. The user requirements dealt with data entry, presentation of results, discussion of results with colleagues and patients, the planning of further diagnostic tests, referral to specialists, and case closure.

### Specific needs of general practice

A CDSS to support diagnoses in the primary care sector should fulfill specific requirements. Physicians criticized alternative systems because they were designed with only rare diseases in mind and did not take into account unusual symptom complexes and the common diseases that play a major role in primary care. Additionally, many such systems in Germany are currently only available for a fee [40]. This may be a barrier, especially for small practices. Furthermore, once a differential diagnosis has been provided, GPs wished for information on how to proceed, such as diagnostic tests or indications to whom patients could be referred. To the best of our knowledge, systems that are available in Germany do not provide information beyond diagnostic suggestions [41].

### Need for transparency

Our results support the findings of other studies that GPs expect a certain transparency when using AI [42, 43]. As previous research has shown, GPs by no means want to be replaced by AI and in some cases fear diminishing capabilities if such systems regularly take over the ‘brainwork’ [14]. GPs are interested in suggestions, inspiration and guidance, but ultimately want to choose and decide themselves [41]. CDSS must address these needs by providing sources for the information they provide.

### Communication with colleagues and patients

The results show that GPs would welcome the chance to communicate with colleagues through the CDSS, which the current software does not allow. In addition to the passive decision support such a system provides, discussions with colleagues often provide important and effective support when making a diagnosis [41]. The integration of a conversation option into the CDSS would make the system significantly more attractive and effective. At the same time, the implementation of such an option would require an extensive technical effort, which may not be possible within the scope of SATURN.

Besides sharing information with colleagues, communication with patients also plays an important role. The GPs discussed whether it would be useful to give patients access to the system and permit them to enter data on, for example, their symptoms themselves, and perhaps to check the accuracy of their data. The literature also emphasizes the importance of involving patients in their own care through interactive tools [18]. However, no consensus was reached on this topic in our workshop. We therefore organized a workshop with patients to explore their points of view, which will be described elsewhere.

### Interface to practice management system

GPs said it was essential to be able to automatically transfer patient data from electronic patient records to the CDSS in order to save time and reduce transmission errors. This requirement has also been mentioned in the existing literature. Sutton et al., for example, argue that CDSS can disrupt workflows if used as stand-alone systems and that poor system integration requiring manual data entry is an important obstacle to the implementation of diagnostic decision support systems [18]. Nurek et al. consider double entry of data as a barrier to the use of CDSS and have therefore requested the integration with electronic patient records [16]. This is a major problem in Germany, as many different providers of practice management systems to manage patient records exist, but mandatory data exchange standards do not [44–46]. Modern standards vary considerably, even though the x-/BDT standard is widely used. However, an interface to link to practice management systems is currently only possible in cooperation with the companies themselves and then only for particular applications. The aim of this project is therefore to develop an initial strategy and to discuss a preliminary solution with system providers. A solution is, however, unlikely to be found in the near term, if data loss is to be prevented [44, 46].

### UCD – benefits and challenges

We consider it useful and feasible to involve GPs as future users in the process. Despite their busy schedules, participants were highly motivated to take part in the project. We believe that the prospect of a useful tool that is specifically designed for their situation increased their willingness to participate. Many ideas emerged on what GPs would like to see in a CDSS. However, when asking what an ‘ideal system’ would look like, it is challenging to prevent GPs from developing unrealistic expectations. In addition to user requirements, technical feasibility and the scope of the project will have to be taken into account during software development. We will therefore prioritize user requirements regarding importance for the GPs and technical feasibility. These prioritized user requirements will serve as a basis in the development of mockups and, subsequently, the first prototype. Hence, the next step is to translate user requirements into system requirements and implement them technically. Mockups and prototypes will be discussed and tested with participants in several iterations.

### Strengths and limitations

Studies with a small sample size are often viewed critically. In qualitative research and in UCD, however, it is inherent in the method that an intensive exchange takes place with a small group of participants. The goal of qualitative methods is not to have a large, statistically representative database but an in-depth examination of individual cases that takes contexts and complexity into account [47]. Especially in UCD, the continuous feedback of participants is important in order to cooperate in shaping and developing the design [48]. In the workshop, the group size of five made constructive discussion possible. Moreover, our sample included both men and women with different amounts of professional experience. In the project, we will closely involve the participants in a number of iterations during system development. Additional participants will be recruited for final usability testing.

The chosen methods (interviews and workshop) were appropriate. By formulating needs and requirements at the start of the UCD process, we ensured the users' points of view were considered before taking the system's perspective. At the same time, it was sometimes difficult to strictly adhere to the syntax rule and we occasionally deviated from it slightly.

A particularly important step in the UCD was to provide participants with the opportunity to express their ideas and needs in a workshop based on an open-ended question. However, it was difficult to ask participants what they would consider an ideal system to look like, while at the same time communicating the limitations inherent in the project.

Some specific results of the requirements analysis may not be transferable to other countries due to differences in work processes. However, our methodological approach can be used and adapted by developers of similar systems when available time and resources are limited. Overall, a multidisciplinary study team proved to be very helpful.

## Conclusions

In order to develop a CDSS for diagnosis in primary care, it proved useful to use interviews and a workshop to conduct requirements analysis, as it enabled us to gain an overview of workflows, information about the user group, their tasks, and essential user requirements. These findings can now be used to design mockups that will be discussed with the users and then implemented as a prototype.

## Supplementary Information

**Additional file 1.** Interview guide.

**Additional file 2.** User requirements list.

**Additional file 3.** Persona. The details in Addfile 3 are for the purposes of illustration only and do not represent the data of any specific individual involved in the study.

## Data Availability

The datasets generated and/or analysed during the current study are not publicly available due to the confidentiality promised to respondents at the time of consent but are available from the corresponding author on reasonable request.

## Abbreviations

AI: Artificial Intelligence
CDSS: Clinical Decision Support System
GP: General practitioner
UCD: User-Centered Design
