# Supplementary material for: Requirements analysis for an AI-based clinical decision support system for general practitioners: a user-centered design process
Source: BMC Med Inform Decis Mak. 2023 Jul 31;23:144. doi: 10.1186/s12911-023-02245-w (PMC10391889; doi:10.1186/s12911-023-02245-w)
Supplement: Supplementary file 1 — Additional file 1. Interview guide. [file 12911_2023_2245_MOESM1_ESM.docx]

# Interviews with General Practitioners

**Research question:** How do general practitioners currently deal with cases of patients with ambiguous symptoms of unknown origin? What tools do they use?

| **Preliminary question** | **Further questions** | **Range of topics** |
| --- | --- | --- |
| Greeting, name + function at the institute.  Thank you very much for taking time for this phone call.  As you already know, the aim of our project is to develop a tool that helps primary care physicians reach diagnoses.  Our conversation today will be about how you proceed with patients who come to your practice with ambiguous symptoms.  Our discussion will last about 30-40 minutes. It is important to understand that there are no right and wrong answers. We would simply like to understand how you proceed in such cases.  Do you have any questions in advance?  I would like to record the interview, but it will be evaluated pseudonymously so that no conclusions can be drawn about your person later. Do you agree to this? *If yes, start recording. If necessary, confirm consent has been given once recording has begun.* |  | Greeting, clarification of the conditions under which the interview is conducted |
| Imagine a patient with ambiguous symptoms or symptoms that don’t match a specific diagnosis.  Maybe diagnostic tests have already been conducted. What would you do? How would you proceed from there? | - Dig deeper, depending on how much information is forthcoming:   - (If applicable.: Perhaps you have a specific case in mind)   - What do you do when the patient is with you?   - What do you do afterwards?   - What sources and aids do you use for support? (e.g., colleagues, books, ebooks, websites or other online-tools, online/offline)   If a search is described at this point, ask questions from the "Use of applications for diagnostics" section!   - How often are you confronted with such a situation? | General approach in cases of diagnostic uncertainty |
| If not already mentioned:  Do you occasionally use digital applications or research options in cases of diagnostic uncertainty?  (If applicable, give an example: Symptom checkers such as Symptoma etc., or search engines, or apps). | - Which do you use? (when several tools are mentioned, ask questions about each of them) - If not, why not? - When several are mentioned - which do you use and what for? - Could you tell me how you proceed?   - What data do you enter?   - For what cases do you use ...?   - Can you tell me when you use it? At what point in time (e.g. during the consultation/after the consultation/between appointments/in consultation with the patient/in consultation with an HCA)?   - If applicable, where (practice, consulting room, at home)?   - How much time do you usually invest in such research?   - What are generally the results of such investigations? (e.g., specific suspected diagnoses, or ideas for further diagnostic tests?)   - What do you then do with that information? / How does the information influence your decision how to proceed?   - When you use …, what device do you usually access it on ...? (PC, smartphone, tablet...) - How much have the results helped you in the past? - What do you feel is missing in …? - Apart from you, who else uses … in the practice? (Or is it generally used by someone else, e.g. the HCA?) - Have you tested any alternatives to …? If so, why did you decide in favor of …? | Use of digital applications in making a diagnosis in cases of diagnostic uncertainty |
| I would also like to talk briefly about rare diseases, i.e. diseases with a very low prevalence (1/2000):  Have you ever had a patient that you diagnosed as having or suspected had such a rare disease? | - How did you reach that conclusion? (research or were you aware of this disease?) - After you had suspected/diagnosed it, what happened then? - Did you refer the patient to someone else? To whom? - How did you find out whom you should turn to? (e.g., colleague, google, SE atlas, other?) - Did you find out what happened to the patient? (was a diagnosis made/or yours confirmed?) | Current approach when faced with rare diseases |
| Is there anything else you would like to say that is relevant to this topic and that we haven’t discussed? |  |  |
| I would like to conclude by asking you a few questions about yourself and your practice. | - Year of birth - Medical specialization - Additional qualifications (or exceptional previous experience, e.g. with rare diseases) - How long have you worked in general practice? (professional experience) - Employed/self-employed - Full-time/part-time? - Size of practice (number of physicians, number of HCAs, other) - Technical equipment:   - What practice management system?   - IT equipment: What devices do you generally use in the practice? (PC, laptop, tablet)   - What devices do you use in your consulting room?   - Internet? Available everywhere? (wlan/lan)   - Apple or Windows? | Sociodemographic data |
| Thank you very much for taking the time for this interview and for providing us with insight into your work. |  |  |
