# Supplementary material for: Requirements analysis for an AI-based clinical decision support system for general practitioners: a user-centered design process
Source: BMC Med Inform Decis Mak. 2023 Jul 31;23:144. doi: 10.1186/s12911-023-02245-w (PMC10391889; doi:10.1186/s12911-023-02245-w)
Supplement: Supplementary file 2 — Additional file 2. User requirements list. [file 12911_2023_2245_MOESM2_ESM.docx]

List of User Requirements

1-50 User Requirements according to Interviews

51-77 User Requirements according to Workshop

| **Task** | **Subtask** | **User Requirement (UR)** | **Prioritized by participants** |
| --- | --- | --- | --- |
| **1. Data entry** | **1.1 Open system** | UR1: The physician needs to be able to use the system in parallel with the practice management software. | N/A |
|  |  | UR10: The physician needs to be able to use the system during patient consultations in the practice. | N/A |
|  |  | UR32: The physician needs to be able to use the system regardless of location (practice, home). | N/A |
|  |  | UR35: The physician needs to be able to use the system on different devices (PC, laptop, tablet, smartphone). | N/A |
|  |  | UR38: The physician needs to be able to use the system at the same time as other people in the practice. | N/A |
|  |  | UR 50: The physician needs to be able to use all functions of the system with different operating systems (Windows, IOS). | N/A |
|  | **1.2 Enter patient data** | UR3/4: The physician needs to be able to enter test results into the system. | N/A |
|  |  | UR6: The physician needs to be able to enter combinations of symptoms into the input mask. | N/A |
|  |  | UR11: The physician needs to be able to enter ambiguous complaints into the system. | N/A |
|  |  | UR25: The physician needs to be able to enter medical terms into the system. | N/A |
|  |  | UR30: The physician needs to be able to enter terms in a known language (German). | N/A |
|  |  | UR65+UR34: The physician needs to be able to indicate diagnoses that have been ruled out in the system. (In the Top 5 booklets, this UR has been prioritized for the subtask ‘Receive results’). | 3 of 5 |
|  |  | UR36: The physician needs to be able to enter data in accordance with data protection rules. | N/A |
|  |  | UR51+UR2: The physician needs to be able to enter symptoms into the system. | 3 of 5 |
|  |  | UR52+UR23: The physician needs to be able to enter suspected diagnoses into the system. |  |
|  |  | UR54+UR21: The physician needs to be able to enter weight, age, gender and height into the system. | 1 of 5 |
|  |  | UR55: The physician needs to be able to enter information on the course of symptoms/how long symptoms have existed into the system . | 0 of 5 |
|  |  | UR56: The physician needs to be able to create patient records in the system. | 3 of 5 |
|  |  | UR57: The physician needs to be able to rework patient cases in the system. |  |
|  |  | UR58: The physician needs to be able to recognize whether, for a suspected diagnosis, all relevant, guideline-recommended tests have been performed. | 0 of 5 |
|  |  | UR59: The physician needs to be able to enter important diagnoses in a patient’s family history into the system. | 0 of 5 |
|  |  | UR61: The physician needs to be able to grant and withdraw authorization for the patient to enter information into the system him- or herself. | 0 of 5 |
|  |  | UR62+UR5: The physician needs to be able to enter laboratory findings into the system. | 0 of 5 |
|  |  | UR63: The physician needs to be able to recognize what additional information should be entered based on the previous entries. | 3 of 5 |
|  |  | UR53: The physician needs to be able to transfer selected socio-demographic and medical data from the practice management software into the system. | 2 of 5 |
| **2. Review results** | **2.1 Receive results** | UR13: The physician needs to have free access to the provided information (including links) within the system. | N/A |
|  |  | UR18: The physician needs to receive suggestions on possible medical diagnoses from the system. | N/A |
|  |  | UR19: The physician needs to be able to retrieve imaging findings from the system. | N/A |
|  |  | UR22: The physician needs to be able to use the system to retrieve information from many different information sources. | N/A |
|  |  | UR33: The physician needs to be able to receive common diagnoses as a result in addition to rare diseases. | N/A |
|  |  | UR49: The physician needs to be able to recognize the prevalence of diagnoses in the system. | N/A |
|  |  | UR64: The physician needs to be able to recognize the differential diagnoses that are under consideration. | 0 of 5 |
|  |  | UR65+UR34: The physician needs to be able to filter out diagnoses that have already been rules out. | 3 of 5 |
|  |  | UR66: The physician needs to be able to recognize how likely a diagnosis is from the system. | 2 of 5 |
|  |  | UR67: The physician needs to be able to recognize from the system which of the displayed diagnoses are ‘red flags’. | 0 of 5 |
|  | **2.3 Evaluate results** | UR14: The physician needs to find regularly updated information in the system. | N/A |
|  |  | UR24: The physician needs to have direct access to the primary sources on which results displayed by the system are based. | N/A |
|  |  | UR27: The physician needs to be able to check the ‘origin’ of the results provided by the system. (transparency of results). | N/A |
|  |  | UR40: The physician needs to receive an indication in the system of when to refer a patient to a specialist. | N/A |
|  |  | UR68+UR15: The physician needs to be able to identify from the system the sources for the information provided. | 2 of 5 |
|  |  | UR69: The physician needs to be able to identify from the system whether a patient case is similar to previous cases. | 0 of 5 |
|  |  | UR58: The physician needs to be able to recognize in the system which examinations are recommended in medical guidelines for a suspected diagnosis. | 0 of 5 |
|  |  | UR66: The physician needs to be able to find out from the system how likely a diagnosis is. | 2 of 5 |
| **3. Discuss results** | **3.1 Discuss results with patient** | UR70: The physician needs to be able to obtain patient information materials from the system. | 0 of 5 |
|  |  | UR71: The physician needs to be able to grant the patient access to read his or her own patient file in the system. | 0 of 5 |
|  |  | UR26: The physician needs to be able receive results as an overview that can be used for a discussion with the patient. | N/A |
|  | **3.2 Communicate with colleagues** | UR75: The physician needs to be able to interact with colleagues on current and closed cases in the system. | 2 of 5 |
| **4. Schedule further diagnostics** |  | UR8: The physician needs to receive recommendations on how to proceed with a diagnostic investigation. | N/A |
|  |  | UR20: The physician needs to receive therapy recommendations from the system. | N/A |
|  |  | UR29: The physician needs to find to medical guidelines in the system. | N/A |
|  |  | UR42: The physician needs to recognize in the system which tests are needed for the diagnosis of rare diseases. | N/A |
|  |  | UR72: The physician needs to be able to recognize in the system how to confirm or rule out the indicated differential diagnoses. | 2 of 5 |
| **5. Refer patient to specialist** | **5.1 Select suitable specialists** | UR7: The physician needs to be able to identify within the system which specialists the patient could be referred to. | N/A |
|  |  | UR73: The physician needs be redirected by the system to existing information platforms (e.g. Center for Rare Diseases, Orphanet). | 1 of 5 |
|  |  | UR74+UR9: The physician needs to be able to identify which institutions could be of help for a suspected diagnosis. | 0 of 5 |
|  | **5.2 Find contact details of suitable specialists** | UR17+UR44: The physician needs to be able to find the contact details of professional colleagues within the system. | N/A |
|  |  | UR45: The physician needs to be able to tell from the system which specialists are located nearby. | N/A |
|  | **5.3 Identify available appointments with specialists** | UR43: The physician needs to be able to recognize in the system when/how promptly patients could be seen by specialists. | N/A |
| **6. Close case** |  | UR76: The physician needs to be able to enter the saved diagnosis in the system after case closure. | 1 of 5 |
|  |  | UR77: The physician needs to be able to view completed cases in the system in an edited form. | 0 of 5 |
