# Supplementary material for: Requirements analysis for an AI-based clinical decision support system for general practitioners: a user-centered design process
Source: BMC Med Inform Decis Mak. 2023 Jul 31;23:144. doi: 10.1186/s12911-023-02245-w (PMC10391889; doi:10.1186/s12911-023-02245-w)
Supplement: Supplementary file 3 — Additional file 3. Persona. The details in Addfile 3 are for the purposes of illustration only and do not represent the data of any specific individual involved in the study. [file 12911_2023_2245_MOESM3_ESM.docx]

## Persona of the ‘General Practitioner’ user group


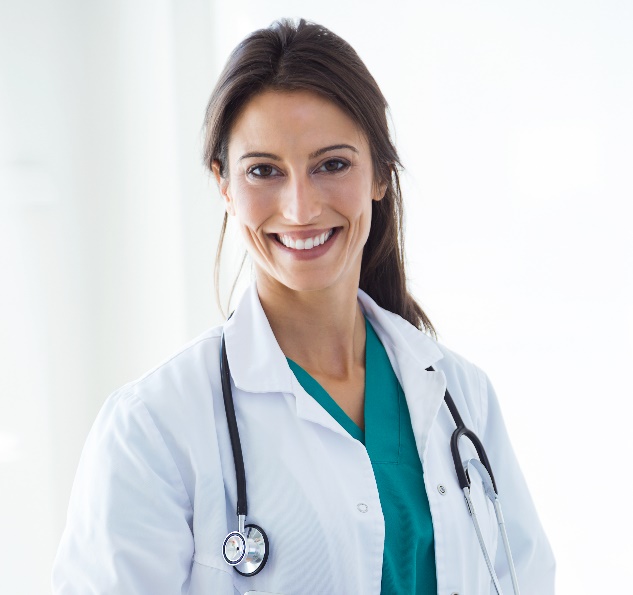


**Dr. med. Lisa Mueller**

Age: 41

Gender: Female

Professional occupation: General practitioner

Lifestyle: tech-savvy, likes to surf on smartphone/tablet

**Work environment**

Ms. Mueller works part-time with her employer and another medical colleague in a general practice. During consultation hours she treats her patients herself. Regular meetings with her colleagues do not take place. In case of questions or diagnostic uncertainty she asks her colleagues for help.

**Professional experience**

Medical experience: she completed specialist GP training at the internal medicine department of a clinic and in a GP practice. She has been employed by a GP practice for 5 years.

Technical knowledge: use of a computer and the patient management system at work, use of various digital devices at home.

**Challenges at work**

Patients occasionally present with general/ambiguous symptoms of unclear origin. In these cases, Ms. Mueller often has a vague idea which area of expertise might be the most appropriate and refers the patient to a specialist. However, in some cases she has no idea what the diagnosis could be. She then lacks the diagnostic resources and the time to thoroughly investigate and follow-up on these cases.

**Existing solutions**

Mrs. Mueller frequently uses the Google search engine and gathers information from various other platforms. Her GP practice has a Deximed account (online information platform for GPs), which she regularly uses to find information. This mainly concerns relatively trivial issues such as the correct dosage of a medication. She has tested several symptom checker tools, but has not yet found a suitable clinical decision support tool for the GP practice.

**Wants and needs**

Mrs. Mueller would like to have a tool that offers her suspected diagnoses based on information about the patient she has entered into the system. She could then discuss the suspected diagnoses with her colleagues.
